# Supplementary material for: Observation of a backward sliding motion for rollers on surfaces in viscoelastic fluid
Source: Nat Commun. 2026 Feb 14;17:2781. doi: 10.1038/s41467-026-69523-9 (PMC13018285; doi:10.1038/s41467-026-69523-9)
Supplement: Supplementary file 1 — Supplementary Information [file 41467_2026_69523_MOESM1_ESM.pdf]

This Supplementary Material contains Supplementary Notes 1−4, Supplementary Figures 1−12.

Table of contents

Supplementary Note 1 2

Supplementary Note 2 5

Supplementary Note 3 6

Supplementary Note 4 7

Supplementary Figure 1 9

Supplementary Figure 2 10

Supplementary Figure 3 11

Supplementary Figure 4 12

Supplementary Figure 5 13

Supplementary Figure 6 14

Supplementary Figure 7 14

Supplementary Figure 8 15

Supplementary Figure 9 15

Supplementary Figure 10 16

Supplementary Figure 11 16

Supplementary Figure 12 17

Supplementary Note 1: Numerical Method

The numerical solver developed in this study comprises two subsolvers: (i) the flow solver, which solves the Navier-Stokes (N-S) equations, and (ii) the viscoelastic solver, which solves the Giesekus constitutive equation.

For fluid solver, we consider an incompressible fluid consists of a solvent and dissolved polymers. The later determines the system's non-Newtonian behavior. Consider the characteristic scales for length $D/2$, time ω^{-1}, velocity $\omega D/2$ and pressure $\omega\eta$, the non-dimensional governing equations for the non-dimensional flow field $\mathbf{u}$ are as follows:

$$\begin{aligned} \nabla \cdot \mathbf{u} &= 0, \\ Re\left(\frac{\partial \mathbf{u}}{\partial t} + \mathbf{u}\cdot\nabla\mathbf{u}\right) &= -\nabla p + \nabla\cdot\mathbf{\Gamma} \end{aligned} \tag{S1}$$

Here ∇ is the non-dimensional gradient operator, t is the non-dimensional time, p is the non-dimensional pressure, Re is the Reynolds number which is defined as $(\omega D^2)/(4\nu)$, $\nu = \eta/\rho$ is the kinematic viscosity, ρ is the fluid density, and $\mathbf{\Gamma}$ is the non-dimensional total stress. The total viscosity η is defined as the sum of the solvent viscosity η_s and polymer viscosity η_p, i.e. $\eta = \eta_s + \eta_p$. Similarly, the total stress $\mathbf{\Gamma}$ is defined as:

$$\mathbf{\Gamma} = \mathbf{\Gamma}_s + \mathbf{\Gamma}_p \tag{S2}$$

Here $\mathbf{\Gamma}_s$ is non-dimensional solvent stress given by the Newtonian law $\mathbf{\Gamma}_s = \beta_s\left[\nabla\mathbf{u} + (\nabla\mathbf{u})^T\right]$ with $\beta_s = \eta_s/\eta$, $\mathbf{\Gamma}_p$ is the non-dimensional polymer stress. For a Newtonian polymer solution, $\mathbf{\Gamma}_p = \beta_p\left[\nabla\mathbf{u} + (\nabla\mathbf{u})^T\right]$ with $\beta_p = \eta_p/\eta$. However, for the viscoelastic (Non-Newtonian) polymer solution considered in

our work, $\mathbf{\Gamma}_p$ needs to be calculated by solving a different constitutive equation via the viscoelastic solver described below.

For viscoelastic solver, we use the dumbbell-based model which considers a polymer chain as a spring attached to two Brownian beads. Considering the small gap and strong shear rate between the roller and wall, we use the following Giesekus constitutive equation[1] to describe the polymer stress $\mathbf{\Gamma}_p$:

$$\mathbf{\Gamma}_p + Wi \overset{\nabla}{\mathbf{\Gamma}}_p + \frac{Wi\alpha}{\beta_p}\mathbf{\Gamma}_p \cdot \mathbf{\Gamma}_p = \beta_p \left(\nabla \boldsymbol{u} + \nabla \boldsymbol{u}^{\mathrm{T}}\right) \tag{S3}$$

where Wi the Weissenberg number, $\overset{\nabla}{\mathbf{\Gamma}}_p$ denotes the Oldroyd's upper convective differentiation, α the mobility parameter which accounts for non-linearity of the model.

We consider a no-slip and no-penetration boundary condition which is enforced at the roller-fluid and wall-fluid interfaces under viscous flow conditions:

$$\begin{aligned} \boldsymbol{u}_B &= \mathbf{0}, \\ \nabla p \cdot \boldsymbol{n}_B &= 0 \end{aligned} \tag{S4}$$

where $\boldsymbol{u}_B$ is the fluid velocity on the roller and wall surface, the subscript B denotes the roller and wall immersed surface, and $\boldsymbol{n}_B$ is the normal vector to the solid surface. The governing equations S1 and S3 with boundary condition S4 are discretized and solved by using OpenFOAM based on finite volume method. To match the experimental results of the rollers reasonably, the following values for the relevant parameters are chosen: $\alpha = 0.2$, $\beta_s = 0.1$, $\beta_s = 0.9$, $\nu = 10^{-5}$ m^2/s. The value of the relaxation time ($\tau = 0.79$s), which determines Wi, is adopted from that measured in experiments for the $c = 0.1$ g/L PAAM solution.

Supplementary Fig. 7a presents the numerical model setup for the sliding motion of a rolling sphere on a flat surface in viscoelastic fluid. The fluid zone reaches $60D$ away from the sphere center in every direction except to the surface (i.e. boundary wall) below, this allows us to ignore any irrelevant boundary effect. The sphere boundary condition is set for a constant angular velocity. When modeling the sliding motion, we set a uniform sliding velocity for the whole fluid zone (including the boundary wall). Supplementary Fig. 7b presents the computational mesh, which is composed of unstructured tetrahedral elements. Far from the roller, the mesh element size is about $10D$. Near the roller (here a semisphere region with radius $1.5D$ above the bounary wall and centered at the point on the boundary which is closest to the roller), the mesh element size is about $D/40$, which is much smaller than that far from the roller.

For the numerical setup of a rolling sphere towards a cliff inside viscoelastic fluid, the fluid zone reaches $60D$ away from the sphere center in all directions (no-slip boundary) except to the no-slip wall below. This wall below has a stair-like geometry with a single step that represents the "cliff". The step height is $2D$. The roller is on the upper part of the wall with a distance d to the edge. The center of the roller has a height $h = 1.15r$ to the upper part of the wall. The mesh resolution near the roller is the same as in Supplementary Fig. 7b.

To validate our numerical methods, we perform two validation cases and compare our results with those in literature. We first consider the creeping flow around a rotating sphere close to a wall in Newtonian fluid, which has been studied theoretically.[2] Supplementary Fig. 8 shows our numerically calculated force F in the horizontal direction (parallel to the wall) and the calculated torque M whose direction is aligned with the angular velocity, which are in good agreement with the previous theoretical results. The definition of F and

M is given by:

$$
\begin{aligned}
F &= (3/2)\pi\eta\omega D^2 \int_{\partial P_0} (-p\boldsymbol{I}\cdot\boldsymbol{n} + \boldsymbol{\Gamma}\cdot\boldsymbol{n})\ \mathrm{d}S \\
M &= \pi\eta\omega D^3 \int_{\partial P_0} (\boldsymbol{\Gamma}\times\boldsymbol{n})\ \mathrm{d}S
\end{aligned}
\tag{S5}
$$

Here $\boldsymbol{I}$ is the unit tensor, $\boldsymbol{\Gamma}$ is the non-dimensional viscous stress acting on the sphere surface, S the non-dimensional sphere surface, $\boldsymbol{n}$ the unit vector along surface normal, ∂P_0 the unit area of the surface. In the second validation case, we have calculated the shear flow induced sphere rotation speed in viscoelastic fluid, following the previous numerical study.[3] Again, our results are in good agreement with the those in literature as shown in Supplementary Fig. 9.

Supplementary Note 2: A comparison between the Giesekus model and the Oldroyd-B model, and the influence of the mobility parameter α

When we choose $\alpha = 0$ in our Eq. (S3), our model reduces to the classic Oldroyd-B model. In Supplementary Fig. 10 we compare the flow field obtained via the Giesekus model and that obtained via the Oldroyd-B model for a roller rotating with $\omega = 1.27$ rad/s and translating with $v = -0.044$ μm/s at a fixed distance $h = 1.15r$ away from the surface. We see that, for both models the streamline exhibits a helical ascent in the y-direction, indicating the occurrence of the rod-climbing effect. This suggest that both models capture the essence of the shear-flow-induced polymer stretch, which explains the backward sliding in our experiments.

Due to the numerical instability of the Oldroyd-B model at high Weissenberg numbers,[4] our simulations are restricted to the Giesekus model with relatively large values of the mobility parameter, such as the $\alpha = 0.2$ chosen

above. This choice partially contributes to the quantitative differences between our simulation and experimental results. However, α has only a minor influence on the onset of backward motion. To demonstrate this, we performed additional simulations with $\alpha = 0.1$ and $\alpha = 0.01$ for rollers at a relatively large $h = 1.4r$ (i.e. a relatively large gap of $0.4r$ between the roller and the ground, to avoid numerical instability). The corresponding k–Wi relation is shown in Supplementary Fig. 11. The data for $\alpha = 0.2$ corresponds to the results presented in Fig. 3d of the main text. As shown in Supplementary Fig. 11, the change of α from 0.2 to 0.01 has slightly increased the Wi_c, and the transition from positive k to negative k becomes a bit sharper. But in general, the effect of different α on the onset of backward motion is relatively small.

Supplementary Note 3: Quantitative analysis of the polymer stretch.

We use the polymer conformation tensor $\boldsymbol{A} = \langle \mathbf{qq} \rangle_{\text{polymer configuration}}$ to describe the intensity of polymer stretch. Here $\mathbf{q}$ is the end-to-end vector of the polymer chain and the angle brackets represent the average over polymer chain configurations. For a polymer in its natural configuration, its end-to-end vector $\mathbf{q}$ has unit length which gives $\mathrm{tr}(\boldsymbol{A}) = 3$. Correspondingly, for a stretched polymer $\mathrm{tr}(\boldsymbol{A}) > 3$ and for a compressed polymer $\mathrm{tr}(\boldsymbol{A}) < 3$. In our model, $\boldsymbol{A}$ is calculated from the polymer stress tensor $\boldsymbol{\Gamma}_p$ via the relation $\boldsymbol{\Gamma}_p = (\beta_p/Wi)\,(\boldsymbol{A} - \boldsymbol{I})$, where $\boldsymbol{I}$ is the unit tensor. The axial ratio of the ellipsoid in Fig. 2a of the main text is then defined as $\mathrm{tr}(\boldsymbol{A})/3$, so that a polymer in its natural configuration (i.e. without stretching or compressing) will be represented by a sphere.

Supplementary Note 4: Force analysis for a rotating and translating sphere near a wall.

In ref.,[5] the motion of a rotating sphere near a wall in Newtonian fluid at low Reynolds number is approximated as a decomposition of a pure rotation and pure translation. Here we show that the same can be done for the rolling motion of a sphere in viscoelastic fluid. To do this, we calculate the parallel component (i.e. parallel to the surface) of the following four forces with our Giesekus model for a pure rotation sphere or a pure translation sphere at a distance $h = 1.15r$ from the surface. Firstly, we calculate the elastic force for a pure rotation sphere, which can be understood as $T_{||}$ in Eq. (1) of the main text. Secondly, the sum of the viscous force and pressure force for a pure rotation sphere, which can be understood as f in Eq. (1) of the main text. Thirdly, the total force for a pure translation sphere, which can be understood as γv in Eq. (1) of the main text. Fourthly, the net force $F_{\mathrm{net}} = f - T_{||} - \gamma v$. The elastic force $T_{||}$ is given as follows:

$$T_{||} = (3/2)\pi\eta\omega D^2 \left(\int_{\partial P_0} (\mathbf{\Gamma}_e \cdot \boldsymbol{n}) \, \mathrm{d}S \right)_{\mathrm{rotation}} \tag{S6}$$

Here $\mathbf{\Gamma}_e = \mathbf{\Gamma}_p - \beta_p(\nabla \boldsymbol{u} + \nabla \boldsymbol{u}^T)$ is the non-dimensional elastic stress acting on the sphere surface, $\mathbf{\Gamma}_p$ is the non-dimensional viscoelastic stress acting on the sphere surface, S the non-dimensional sphere surface, $\boldsymbol{n}$ the unit vector along surface normal, ∂P_0 the unit area of the surface. Similarly, f is given as:

$$f = (3/2)\pi\eta\omega D^2 \left(\int_{\partial P_0} (-p\boldsymbol{I} \cdot \boldsymbol{n} + \mathbf{\Gamma}_v \cdot \boldsymbol{n}) \, \mathrm{d}S \right)_{\mathrm{rotation}} \tag{S7}$$

γv is given as:

$$\gamma v = (3/2)\pi\eta\omega D^2 \left(\int_{\partial P_0} (-p\boldsymbol{I} \cdot \boldsymbol{n} + \boldsymbol{\Gamma}_v \cdot \boldsymbol{n} + \boldsymbol{\Gamma}_e \cdot \boldsymbol{n}) \, \mathrm{d}S \right)_{\text{translation}} \tag{S8}$$

Here $\boldsymbol{\Gamma}_v = (\beta_p + \beta_s)(\nabla \boldsymbol{u} + \nabla \boldsymbol{u}^T)$ is the non-dimensional viscous stress acting on the sphere surface. Supplementary Fig. 12 presents the numerically calculated $T_{||}$, f, γv and F_{net} as a function of Wi. Clearly, at small Wi, $T_{||}$ is smaller than f. This suggests a forward motion which corresponds to $\gamma v < 0$. When Wi becomes greater than a critical value Wi_{c}, $T_{||}$ becomes larger than f. This suggests backward motion which corresponds to $\gamma v > 0$. Remarkably, F_{net} is almost zero for all Wi considered here. This validates the motion decomposition strategy mentioned above and also validates Eq. (1) of the main text. Note that f becomes smaller as Wi increases. This is a sign of shear thinning which is expected for the Giesekus model. Even though such shear thinning effect facilitates the backward motion, the major driving force for the backward motion in our experiments is $T_{||}$ which is clearly revealed in Fig. 2c of the main text and in Supplementary Video 5.

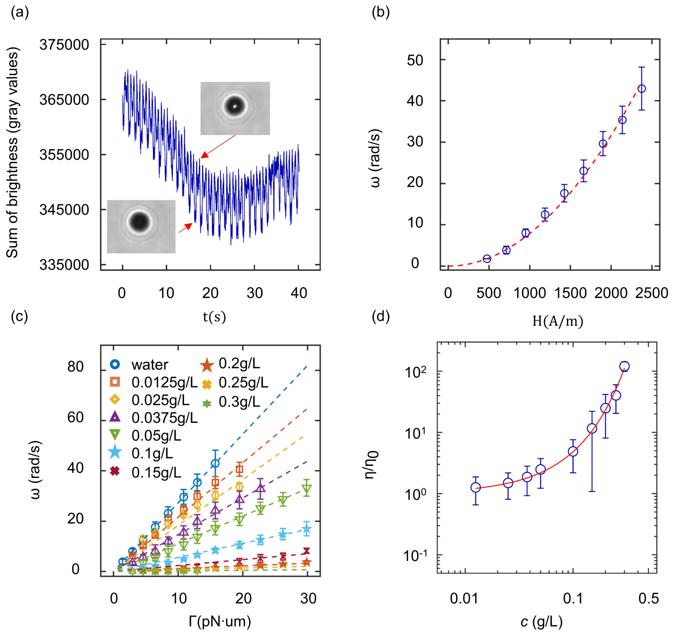

Supplementary Fig. 1 Colloid rotation determination, magnetic torque calibration and polymer viscosity measurement. A rotating magnetic field $\mathbf{H}(t)$ in the xz plane was generated with $H_x(t) = H\cos(\omega_H t)$ and $H_z(t) = H\sin(\omega_H t)$. The field frequency was fixed at $\omega_H = 20\pi$ s^{-1}. Janus superparamagnetic colloidal spheres ($D = 4.5$ μm) were prepared as described in the *Methods* section of the main text and were dispersed in water. **a,** The experimentally measured light intensity variation within a 50×50 pixel^2 (or 7.4×7.4 μm^2) area under the microscope where a Janus superparamagnetic colloidal sphere is rolling. With a Fourier analysis we determined the rotation period of the roller to be 1.36 second. This corresponds to a rolling angular velocity of $\omega = 4.64$ rad/s. A magnetic field of $H = 711.4$ A/m was applied to achieve the rolling here. **b,** The measured angular velocity ω of the Janus colloidal sphere rollers in water as a function of the applied magnetic field strength H. Experimental data are shown as circles, and the solid line is a fit to the relation $\omega = k_m H^2$, yielding $k_m = (7.90 \pm 0.3) \times 10^{-6}$ m^2/(A$^2\cdot$ s). Considering the viscosity of water $\eta_0 = 0.0013 \pm 0.0002$ Pa·s, this gives $\gamma_m = \pi\eta_0 D^3 k_m = 2.94 \times 10^{-6}$pN·$\mu$m·A^{-2}·m^2. Thus we have calibrated the magnetic torque applied to the sphere $\Gamma = \gamma_m H^2$. **c,** The experimentally measured ω-Γ relation for magnetic particles in PAAM solutions of varying concentrations. Dashed lines are the linear fittings to $\omega = \Gamma/(\pi\eta\, D^3)$ which gives the effective viscosity η of the PAAM solutions. **d,** The measured η as a function of c for PAAM solutions at $c < 0.3$ g/L. The results fit (solid line) nicely to an exponential function $\eta/\eta_0 = \exp(\beta c)$, with fitted $\beta = 15.8$ L/g. All error bars represent standard deviations obtained from five independent measurements. Source data are provided as a Source Data file.

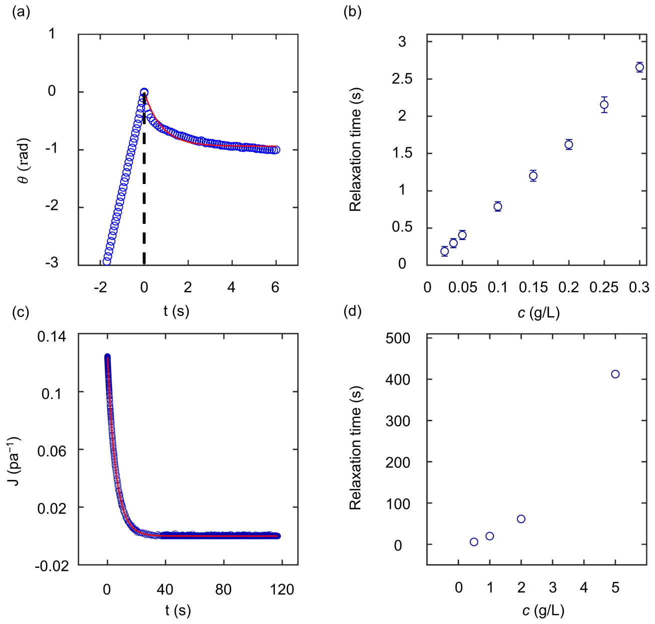

Supplementary Fig. 2 Relaxation time measurement for PAAM solution. Detailed description of the measurement is found in the *Methods* section of the main text. **a,** Data points (blue circles) are the angular displacement θ of a colloidal trimmer as a function of time. The trimer is rotating in a 0.1 g/L PAAM solution with fixed angular velocity at $t < 0$ and the magnetic torque is suddenly removed at $t = 0$. The $t > 0$ part shows the angular relaxation of the colloid trimer, from which we determine the relaxation time τ of the PAAM solution via fitting (the red solid line) to an exponential function $\theta(t) = a(e^{-t/\tau} - 1)$, with fitted $a = 0.94$ rad and $\tau = 0.78$ s here. This method is used for all the dilute and semidilute PAAM solution and the measured τ is not sensitive to the initial angular velocity of the trimer. **b,** The measured relaxation time τ as a function of polymer concentration c in the dilute and semidilute regime. **c,** The data points (SCblue circles) shows the relaxation of the compliance J when the applied stress is removed at $t = 0$ in the creep-recovery experiments (via a rotational rheometer).[6] The relaxation time τ of the PAAM solution is determined by fitting (red solid line) the data points to $J(t) = J_0(e^{-t/\tau})$, with $J_0 = 0.124$ Pa^{-1} and fitted $\tau = 5.7$ s for a 0.5 g/L PAAM solution here. **d,** The measured relaxation time as a function of polymer concentration c in the high-concentration regime. All error bars represent standard deviations obtained from five independent measurements. Source data are provided as a Source Data file.

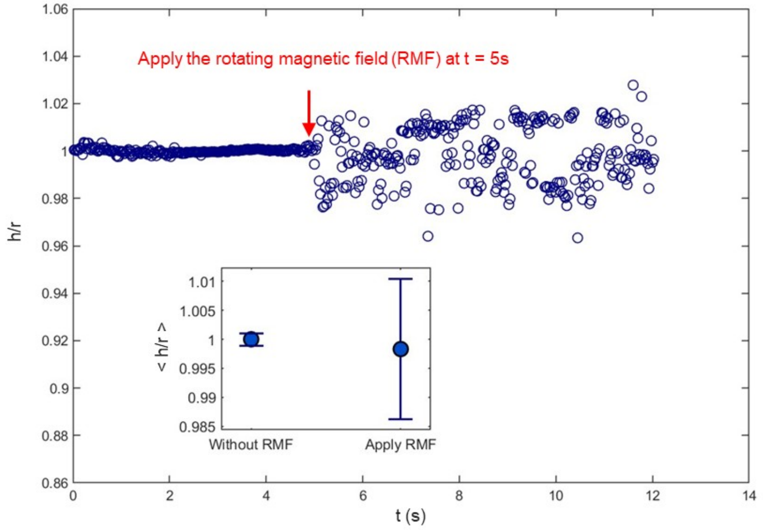

Supplementary Fig. 3 Measurement of the gap distance between the millimeter roller and the ground. The measured distance h between the center of a roller ($D = 2r = 2$ mm) and the ground (glass substrate) as a function of time t. h is measured via a side-view video of the roller (see Supplementary Video 6) captured with a CCD camera through a lens. The roller is immersed in a 5 g/L PAAm solution. At $t < 5$ s, no magnetic field is applied, therefore the roller remains in contact with the ground (i.e. $h = r$). At $t = 5$ s, an 8-Hz rotating magnetic field is applied, this causes the roller to perform backward sliding motion (see Supplementary Video 6) and its h to fluctuate. The measured $h = 0.999 \pm 0.012r$. The error bar (which includes both the fluctuation of the roller's height and the uncertainty in its profile detection) is the standard deviation of the measured h at different $t > 5$ s. Note that the measured mean value of h deviates very little ($< 0.001r$) from r. Therefore it is reasonable to use the error bar as an upper limit for the mean gap distance between the roller and the ground, which is about $0.012r$, or 12 μm for the $D = 2$ mm roller. Source data are provided as a Source Data file.

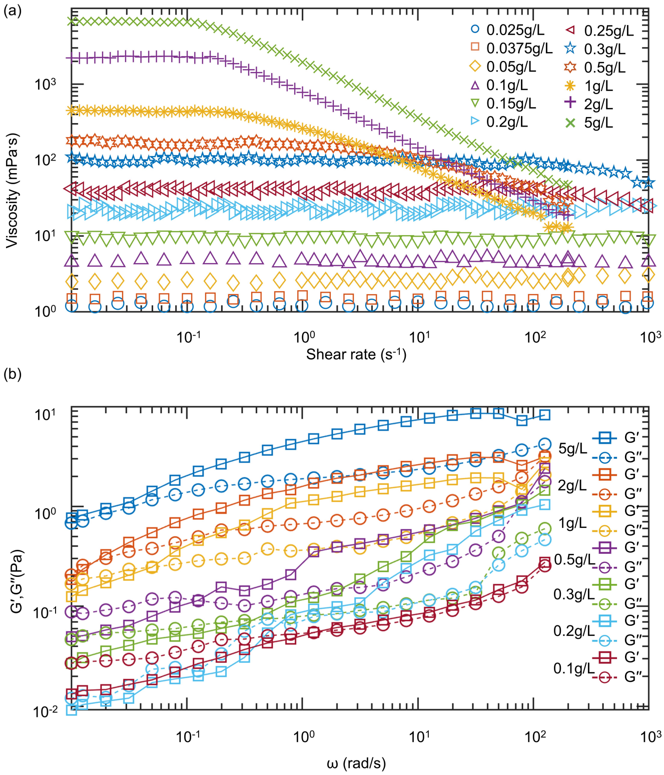

Supplementary Fig. 4 Rheological properties of PAAM solutions in our work. a, The viscosity of PAAM aqueous solutions at various PAAM concentrations c was measured using a rotational rheometer as a function of shear rate. Shear thinning is negligible when c $\leq$ 0.3 g/L. It becomes relevant for the range of shear rate in our experiments when c $>$ 0.3 g/L. **b,** The storage modulus G' and loss modulus G'' of polyacrylamide (PAAM) aqueous solutions at different concentrations were measured using a rotational rheometer as a function of oscillatory angular frequency. Source data are provided as a Source Data file.

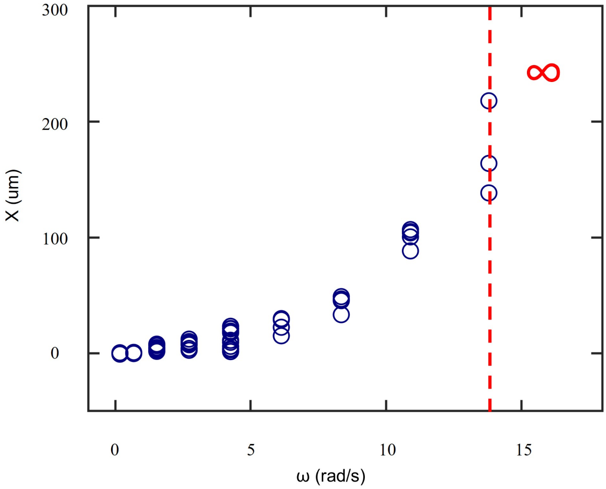

Supplementary Fig. 5 Evidence of rolling-induced roller-to-surface attraction. Due to the effective attraction between the roller and the surface, the roller can roll at the ceiling for a certain distance before it drops to the ground under gravitational pulling. The figure here shows the measured displacement X on the ceiling as a function of the rotation speed ω of a $D = 4.5$ μm roller in a $c = 0.05$ g/L PAAM fluid. Clearly, X rapidly increases with ω, indicating a stronger roller-to-surface attraction at large ω. In addition, when $\omega > 15$ rad/s, the attraction between the roller and the surface become so strong that the roller no longer drop to the ground in our experiments. Note that the polymer in the viscoelastic fluid can induce a depletion attraction $U_{\rm depletion}$ between the roller and the ceiling. We estimate its value via the Asakura-Oosawa theory.[7] Consider the polymer (PAAM) radius of gyration $R_{\rm g} = 0.0158 M_{\rm w}^{0.58}$ nm,[8] number density $n = N_{\rm A} c/M_{\rm w}$, and the distance between the sphere surface and the wall $z < 2R_{\rm g}$. This gives $U_{\rm depletion} = -2\pi R_{\rm g}^2 D n k_{\rm B} T$ at $z = 0$. In our experiment, the molecular weight $M_{\rm w} = 18$ MDa. This gives $U_{\rm depletion} = -3.08 k_{\rm B} T$ for $c = 0.05$ g/L. This depletion attraction strength is not sufficient to hold the roller to the ceiling for a prolonged period of time, considering the buoyant weight 286 fN of the $D = 4.5$ μm roller. This is confirmed in our experiments where the roller falls quickly from the ceiling when the ω of the roller is small. Source data are provided as a Source Data file.

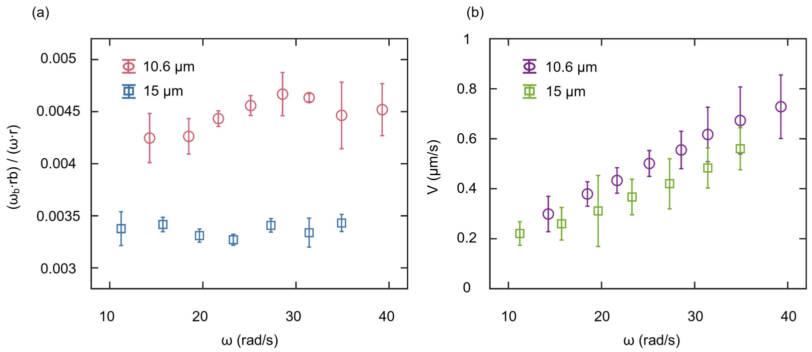

Supplementary Fig. 6 Motion transmission from magnetic sphere to non-magnetic sphere. The counterclockwise velocity of the superparamagnetic colloidal spheres and the SiO_2 sphere (diameters of 10.6 μm and 15 μm respectively) as a whole at different angular velocities of the superparamagnetic colloidal sphere in the 0.1 g/L PAAM solution. **a,** The experimentally measured motion transmission ratio $\omega_b r_b/\omega r$ as a function of ω. Here ω_b and r_b the angular velocity and radius of the non-magnetic sphere respectively, ω and r the angular velocity and radius of the magnetic sphere. The results indicate that the motion transmission ratio is not sensitive to the angular velocity ω of the magnetic sphere in the range of ω considered. **b,** The orbital speed V of the non-magnetic spheres as a function of ω for the circular motion of the non-magnetic sphere as described in Fig. 4b of the main text. V increases almost linearly with ω. All error bars represent standard deviations obtained from five independent measurements. Source data are provided as a Source Data file.

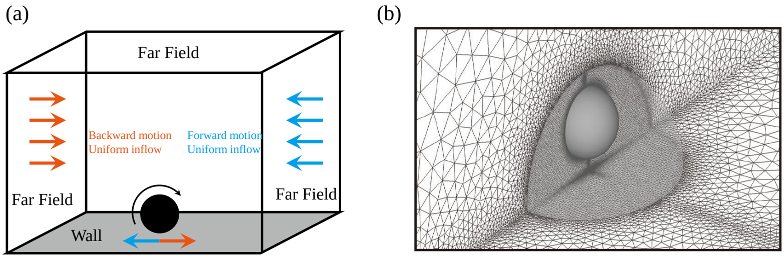

Supplementary Fig. 7 Numerical setup and mesh setup around roller. (a) The numerical setup. The fluid zone is inside a square box of $120D \times 120D$ base area and $60D$ by height. The sphere sits above the center of the base with a fixed sphere-to-wall gap distance h. (b) The mesh setup. The computational mesh is composed of unstructured tetrahedral elements. Far from the roller, the mesh element size is about $10D$. Near the roller (here a semisphere region with radius $1.5D$ above the bounary wall and centered at the point on the boundary which is closest to the roller), the mesh element size is about $D/40$.

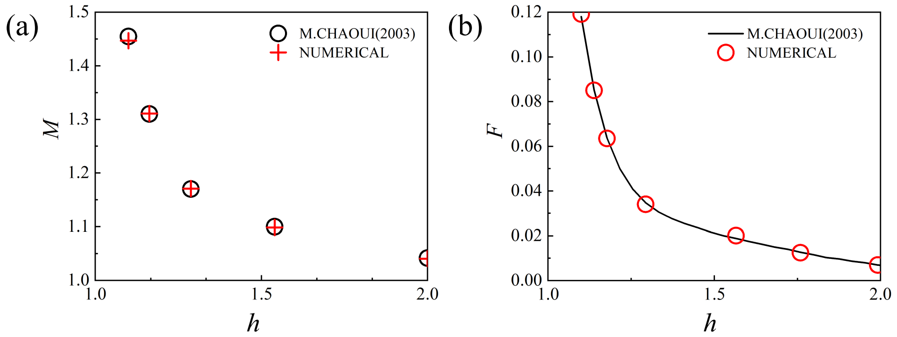

Supplementary Fig. 8 Validation case 1: Creeping flow around a rotating sphere close to a wall(rotating axis is parallel to the wall). (a) The numerically calculated torque (unit: $\pi\eta\omega D^3$) as a function of h. (b) The numerically calculated force F (unit: $(3/2)\pi\eta\omega D^2$) experienced by the sphere in the direction parallel to the wall as a function of h. We set the Reynold number to be $Re = 0.01$ and compare our results with the data in.[2] Source data are provided as a Source Data file.

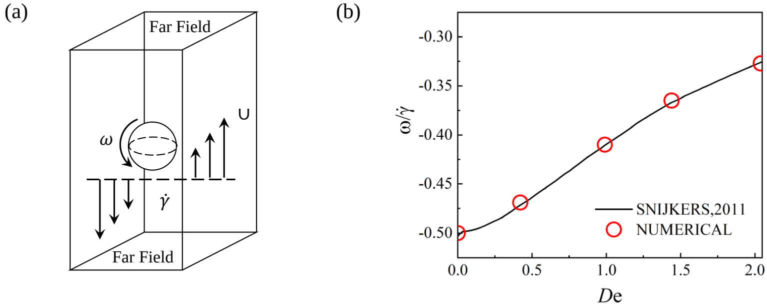

Supplementary Fig. 9 Validation case 2: Shear flow induced rotation for a sphere in Non-Newtonian fluid. (a) Schematic diagram of the case, ω is the angular velocity of the sphere at steady state, $\dot{\gamma}$ is the shear rate, U is the boundary flow velocity. (b) Our numerically calculated $\omega/\dot{\gamma}$ as a function of the Deborah number $De = \tau\dot{\gamma}$, in comparison with the data in.[3] We have set $Re = 0.01$ and therefore ignored the inertial effect. Source data are provided as a Source Data file.

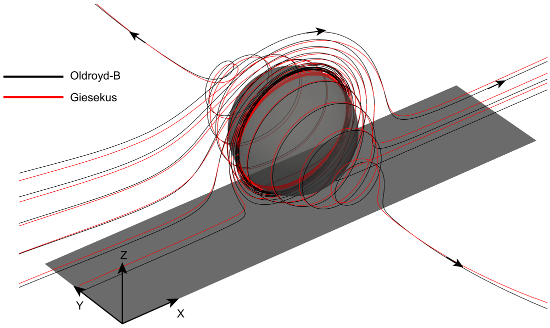

Supplementary Fig. 10 A comparison of the flow field calculated with the Oldroyd-B model and the Giesekus model.

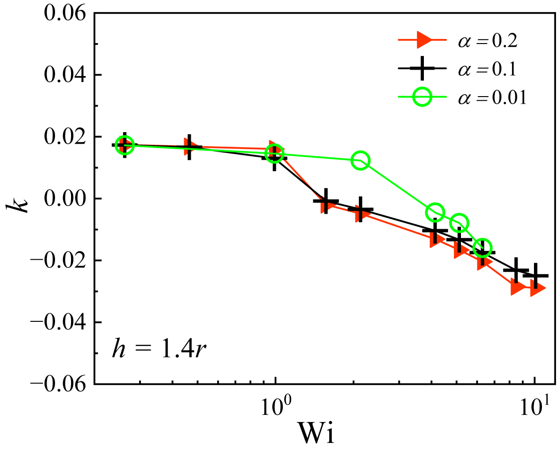

Supplementary Fig. 11 The influence of the mobility parameter α in the k-Wi relation. $h = 1.4r$ is chosen. The curve for $\alpha = 0.2$ here corresponds to the same one in Fig. 3d of the main text. Source data are provided as a Source Data file.

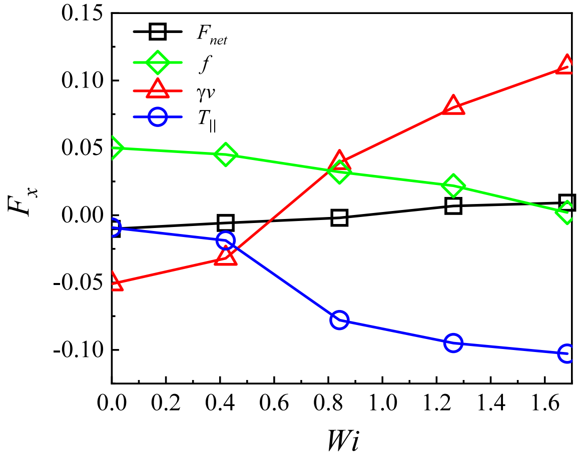

Supplementary Fig. 12 Data points are the numerically calculated force $T_{\|}$, f, γv and $F_{\rm net}$ (unit: $(3/2)\pi\eta\omega D^2$) at different Wi as mentioned in Supplementary Note 4. Here $\omega = 1.27$ rad/s, Wi is adjusted by changing relaxation time τ. Lines are guides for the eye. Source data are provided as a Source Data file.

References

[1] Giesekus, H. A simple constitutive equation for polymer fluids based on the concept of deformation-dependent tensorial mobility. *J. Non-Newtonian Fluid Mech.* **11**, 69–109 (1982).

[2] Chaoui, M. & Feuillebois, F. Creeping flow around a sphere in a shear flow close to a wall. *Q. J. Mech. Appl. Math.* **56**, 381–410 (2003).

[3] Goyal, N. & Derksen, J. J. Direct simulations of spherical particles sedimenting in viscoelastic fluids. *J. Non-Newtonian Fluid Mech.* **183**, 1–13 (2012).

[4] Keunings, R. On the high Weissenberg number problem. *J. Non-Newtonian Fluid Mech.* **20**, 209–226 (1986).

[5] Goldman, A. J., Cox, R. G. & Brenner, H. Slow viscous motion of a sphere parallel to a plane wall—I: Motion through a quiescent fluid. *Chem. Eng. Sci.* **22**, 637–651 (1967).

[6] Koser, A. E. et al. Measuring material relaxation and creep recovery in a microfluidic device. *Lab Chip* **13**, 1850–1853 (2013).

[7] Asakura, S. & Oosawa, F. On interaction between two bodies immersed in a solution of macromolecules. *J. Chem. Phys.* **22**, 1255–1256 (1954).

[8] Kubota, K. et al. Characterization of poly(N-isopropylmethacrylamide) in water. *Polym. J.* **22**, 1051–1057 (1990).
