# Supplementary material for: Observation of a backward sliding motion for rollers on surfaces in viscoelastic fluid
Source: Nat Commun. 2026 Feb 14;17:2781. doi: 10.1038/s41467-026-69523-9 (PMC13018285; doi:10.1038/s41467-026-69523-9)
Supplement: Supplementary file 2 — Description of Additional Supplementary Files [file 41467_2026_69523_MOESM2_ESM.pdf]

Supplementary movie 1:

The motion of rollers in two different viscous fluids and in three different viscoelastic fluids as indicated in the movie. The D = 4.5 μm roller is used here except in the case of the egg white liquid where a cylindrical magnet with diameter 2 mm and length of 5 mm is used. The rotating axis of all rollers point to the same direction (i.e. the positive y direction as shown in Fig. 1a of the main text), with the given angular velocity ω as indicated in the movie. All rollers in viscoelastic fluid move from right to left (i.e. backward sliding) while those in viscous fluid move from left to right.

Supplementary movie 2:

The motion of different sized and shaped rollers on flat as well as on rough surfaces in the PAAM solution (viscoelastic fluid). The size and shape of the rollers, their angular velocity ω and the PAAM concentration are indicated in the movie. All rollers reveal backward sliding.

Supplementary movie 3:

The motion of a D = 4.5 μm colloidal particle and a colloidal trimer roll in a 0.025 g/L PAAM solution with k = 0. The angular velocity of the rollers are indicated in the movie.

Supplementary movie 4:

The flow field around a rolling D = 30 μm magnetic colloidal particle in a 0.1 g/L PAAM solution, revealed via small tracer particles (D = 1 μm polystyrene beads). The angular velocity of the roller is ω = 1.27 rad/s. Note that the small tracer particles have nonzero migration towards both positive and negative y-axis. This agrees with the numerically calculated flow field in Fig. 2a of the main text.

Supplementary movie 5:

The backward sliding of a micrometer roller (D = 4.5 μm) and a millimeter roller (D = 2 mm) towards cliffs in viscoelastic fluids. Both rollers does not roll off the cliff (i.e. their translational motion ceases when they arrive at the cliff), even though they are

continuously rotating. For the micrometer roller, the cliff is the side wall of a 15 μm deep square pit. The profile of a cross section (in x-z plane) of the square pit is shown, which is measured using a surface profilometer (Alpha-step D-600, KLA Corporation, USA). For the millimeter roller, the cliff is constructed with a stack of 4 glass slides of about 1.2 mm thick. Note that the millimeter roller stops the backward sliding motion when it arrives at the edge of the cliff, even though it is still rotating.

Supplementary movie 6:

Side-view video of a D = 2 mm roller on glass substrate inside PAAm solution with c = 5 g/L. The video is captured with a CCD camera through a lens (75 mm focal length). An 8-Hz rotating magnetic field is applied at t = 5 s, which drives the roller to roll. We have switched the rotating direction of the magnetic field so that the roller remains in the field of view during the period of the video. The left hand side shows the raw video. To estimate the fluctuation of the roller's height h as shown in
Supplementary Fig. 3, we labeled the ground and the profile of the roller as shown in the right hand side. The profile of the roller is obtained via fitting the gray-scale image of the roller to a circle of radius 1 mm.

Supplementary movie 7:

Experimental evidence of roller-wall attraction. (top) The rolling of D = 4.5 μm magnetic particles at the ceiling of a sample cell, where the microscope objective is focused on. At the beginning of the video, a colloidal sphere at the left side is rolling on the ceiling (i.e. in focal 20 plane) towards right. At a later time it falls (i.e. gets out of focus) towards the bottom surface of the sample cell and starts to roll towards left. (bottom) The rolling of D = 4.5 μm colloidal particles and trimmers along the side wall of a 15μm deep square pit with 80μm×80μm size. The applied magnetic field $H_x = H\cos \omega_H t$, $H_y = -H\sin\omega_H t$, $H_z = 0$ with $H = 1257.2$ A/m and $\omega_H = 200\pi s^{-1}$ so that the colloid rotate along the z axis (i.e. perpendicular to the field of view) as indicated in Fig. 1a of the main text.

Supplementary movie 8:

The circular motion of non-magnetic particles induced by the rotation of the attached small magnetic particles in 0.1 g/L PAAM solution. The magnetic particles rotate along the z axis with their angular velocities indicated in the movie.

Supplementary movie 9:

Demonstration of cargo delivery towards the target location via the protocols defined in Fig. 4c of the main text.
